# Mu-opioid receptor activation potentiates excitatory transmission at the habenulo-peduncular synapse

**DOI:** 10.1016/j.celrep.2025.115874

**Published:** 2025-06-19

**Authors:** Sarthak M. Singhal, Agata Szlaga, Yen-Chu Chen, William S. Conrad, Thomas S. Hnasko

**Affiliations:** 1 Department of Neurosciences, University of California, San Diego, La Jolla, CA 92093, USA; 2 Research Service, Veterans Affairs San Diego Healthcare System, San Diego, CA 92161, USA; 3 Lead contact

## Abstract

Identifying how opioids modulate signaling in relevant neurocircuitry is essential for developing new therapeutic strategies for opioid addiction. The medial habenula (MHb) is a mu-opioid receptor (MOR) hotspot that projects predominantly to the interpeduncular nucleus (IPN); however, little is known about MOR function in this pathway. Using reporter mice, we observed MOR expression in a subset of MHb and IPN neurons, where its activation induces inhibitory effects on neuronal activity. However, stimulation of MOR^+^ axons at the habenulo-peduncular (HP) synapse leads to excitatory currents that are significantly potentiated by MOR agonism. These facilitatory effects were also observed at cholinergic-defined HP synapses, depend on a monosynaptic mechanism, and are disrupted by genetic disruption of MOR in the presynaptic MHb. Thus, MORs induce a canonical inhibitory effect in somatodendritic compartments but non-canonical facilitatory effects on evoked glutamate transmission at the HP synapse, establishing a distinct mode by which MORs can modulate neuronal function.

## In brief

Singhal et al. demonstrate the physiological role of mu-opioid receptor (MORs) in the medial-habenula-to-interpeduncular-nucleus (HP) pathway. Post-synaptic MORs canonically inhibit medial habenula and interpeduncular nucleus activity, but presynaptic MORs non-canonically facilitate excitatory transmission at HP synapses, establishing a distinct mode by which MORs can modulate neuronal function.

## Graphical abstract

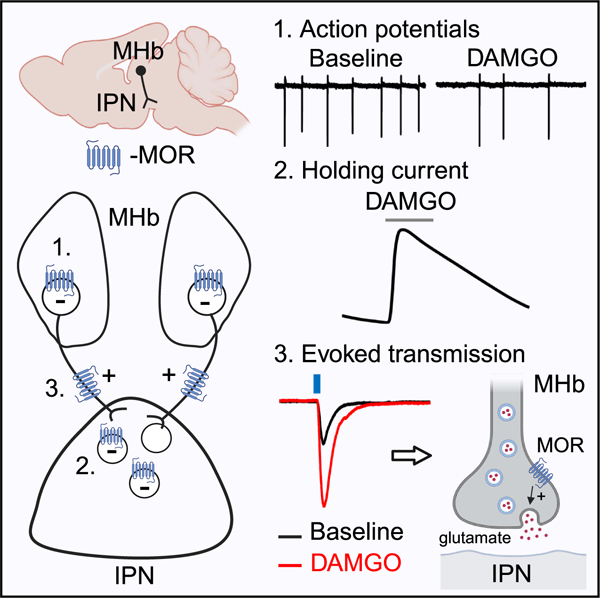

## INTRODUCTION

The opioid addiction crisis poses a huge burden on public health. Relapse commonly occurs after cessation of drug use, in part due to the onset of opioid withdrawal symptoms.^1,2^ The locus coeruleus and mesolimbic system have been implicated in regulating opioid withdrawal symptoms,^3,4^ however, mu-opioid receptors (MORs) are expressed widely. To open new avenues for therapeutic intervention, there is a clear need to identify how other neural systems contribute.

The medial habenula (MHb) is a bilateral, epithalamic structure that is part of the habenular complex and expresses one of the highest densities of MORs.^5–8^ It is primarily glutamatergic but also contains neurons that co-synthesize either acetylcholine or the tachykinins substance P and neurokinin B.^9,10^ The MHb receives inputs from septal nuclei and sends dense projections to the interpeduncular nucleus (IPN) through the fasciculus retroflexus, comprising the habenulo-peduncular (HP) circuit.^11^ MHb projections to the IPN are topographically organized with dorsal MHb neurons projecting mainly to rostral (rostral IPN [IPR]) and lateral (lateral IPN [IPL]) subregions of the IPN and ventral MHb neurons sending projections to IPR and central subregions.^12^ The IPN is a midline midbrain nucleus composed primarily of GABAergic neurons and sends efferents to the raphe and tegmental nuclei, among others.^13,14^

The HP circuit is important in regulating nicotine dependence and withdrawal and is more generally implicated in the regulation of anxiety and mood.^11,12,15–17^ For example, hyperactivity of the HP circuit increased depression-like behaviors in rats,^18^ and GABA_B_ and CB1 receptor actions at the HP synapse regulated fear behavior and the expression of aversive memory.^19,20^ Recent behavioral pharmacology studies also revealed a role for MHb MOR expression in opioid dependence. Deletion of MORs from β4 nicotinic receptor expressing cells, which are enriched in MHb, attenuated the affective and physical signs of morphine withdrawal^21^ and impaired social behaviors.^22^ Optogenetic stimulation of habenular MOR projections triggered avoidance and despair-like behavior.^23^

MORs are G_i_-linked G-protein coupled receptors (GPCRs), and their activation can inhibit cyclic AMP (cAMP) production, open inward rectifying potassium channels, and thereby make neurons less excitable. MORs also localize to axon terminals where they can inhibit synaptic transmission.^24–26^ However, it is unknown how MOR activation affects excitability or neurotransmission specifically within the HP circuit. Understanding the consequences of MOR signaling in the HP circuit is a key step toward revealing its role in opioid addiction.

Here, we used reporter mice and *in situ* hybridization to visualize MOR expression in a subset of MHb and IPN neurons and patch-clamp electrophysiology to reveal how MOR activation influences MHb and IPN neurons and synapses. We found that MORs have canonical effects, inhibiting MOR-expressing neurons in both MHb and IPN. In contrast, we found that MOR activation markedly potentiated evoked excitatory transmission at the HP synapse, including from glutamate-releasing HP synapses made by cholinergic neurons. Finally, we provide pharmacological and genetic evidence indicating that that these facilitatory effects on neurotransmission are due to the direct activation of presynaptic MORs at the HP synapse. To our knowledge this is the first report of a direct facilitatory effect of MOR activation on neurotransmission, suggesting a new non-canonical mode of MOR signaling. In sum, we demonstrate compartment-specific effects of MOR activation on neuronal excitability and excitatory transmission in the HP circuit and provide the first electrophysiological characterization of the physiological consequences of MOR in this circuit that is of known importance to aversion, anxiety, and addiction.

## RESULTS

### MOR is expressed in MHb and IPN neurons and its activation is inhibitory

**Figure 1.**
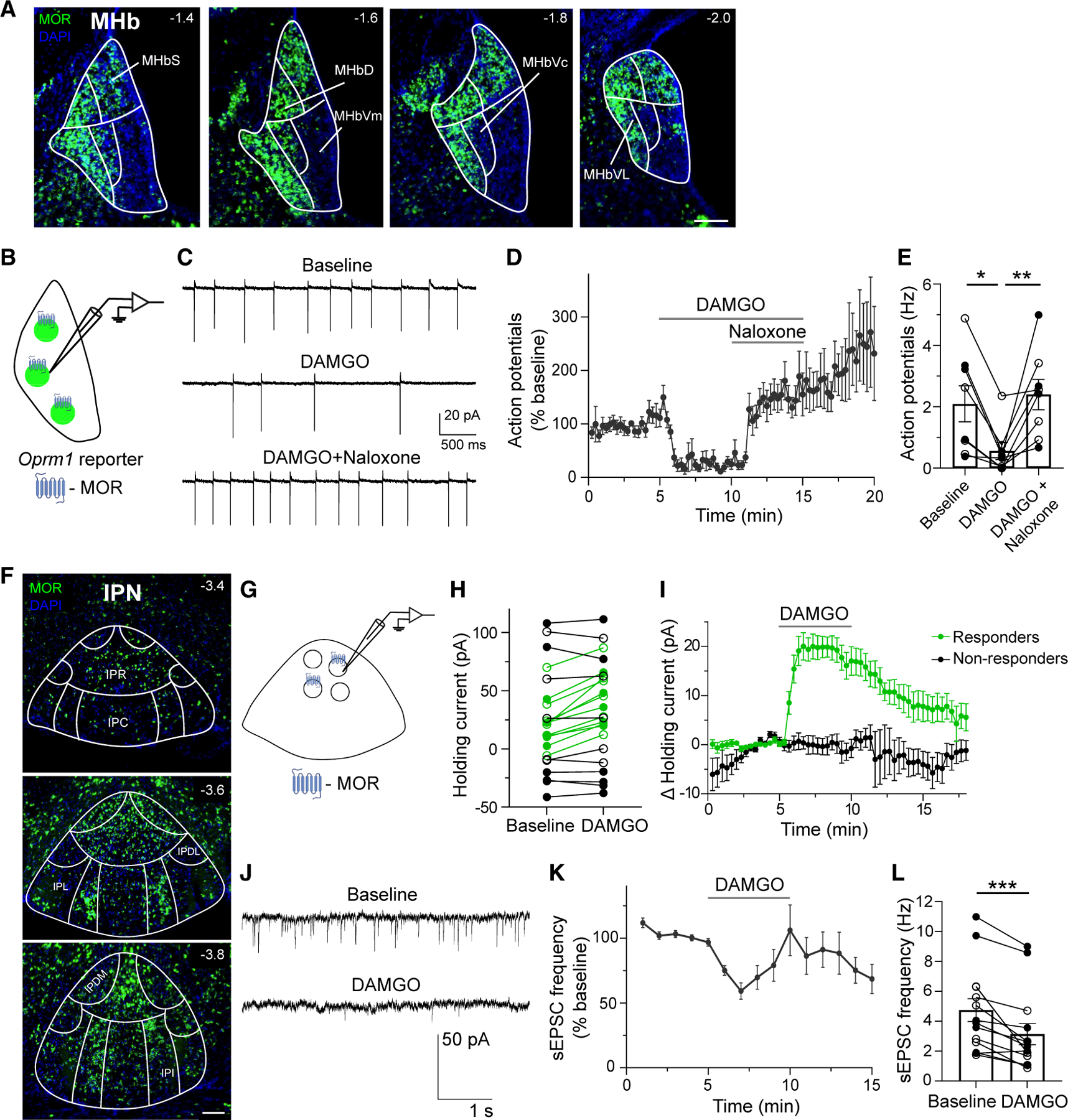
Inhibitory effects of MOR activation in the MHb and IPN (A) Example images through MHb show ZsGreen reporter expression (green) resulting from MOR-Cre; DAPI in blue. MHb subregions: S, superior; D, dorsal; Vc, ventral central; VL, ventral lateral; Vm, ventral medial. Scale bar, 100 μm. (B) Schematic of cell-attached recordings from MOR^+^ cells in the MHb obtained from ZsGreen reporter mice. (C–E) Example recording (C), averaged time trace (D) and bar graph (E) of cell-attached recordings showing DAMGO (5 μM)-mediated inhibition of action potentials, which was reversed by naloxone (5 μM) application (*n* = 8 cells/4 mice); Friedman test, *p < 0.05, **p < 0.01. (F) Representative images through IPN show ZsGreen reporter expression (green) resulting from MOR-Cre; DAPI in blue. IPC, caudal; IPDL, dorsal lateral; IPDM, dorsal medial; IPI, intermediate; IPL, lateral; IPN subregions: IPR, rostral. Scale bar, 100 μm. (G) Schematic of whole-cell recordings from untagged IPN cells, some of which express MOR. (H) DAMGO (1 μM) application increased holding current in a subset of IPN neurons (*n* = 12/23), pseudo-colored green, consistent with postsynaptic expression of MORs in a subset of IPR neurons. Non-responders are colored black. (I) Averaged time traces show DAMGO-induced outward current in responding (*n* = 12) versus non-responding neurons (*n* = 11); *n* = 21 mice. (J–L) Example recording (J), averaged time trace (K), and bar graph (L) of whole-cell recordings showing DAMGO (1 μM)-mediated inhibition of sEPSC frequency in IPN neurons (*n* = 14 cells/13 mice); Wilcoxon test, ***p < 0.001. Solid and open circles showing individual neurons (E, H, and L) represent data from male and female mice, respectively. Data with error bars represent mean ± SEM.

To investigate the expression of MOR in neurons comprising the HP circuit, we generated mice that express a fluorescent reporter in cells that expressed MOR (MOR-Cre). Within the MHb, reporter expression was observed in a subset of MHb neurons concentrated in lateral and dorsal subregions across its rostral to caudal extent (Figure 1A). We next used fluorescent *in situ* hybridization by RNAscope to validate this expression pattern. We used probes targeting *Oprm1* (MOR), as well as a glutamatergic marker (vesicular glutamate transporter 2 [VGLUT2] *Slc17a6*), and a cholinergic marker (choline acetyltransferase [*Chat*]). As expected, mRNA encoding the MOR was concentrated in lateral and dorsal MHb subregions (Figure S1A), where it partially colocalized with cholinergic and glutamatergic markers (Figure S1B).

We next used patch-clamp electrophysiology (cell-attached recordings) to test how MOR activation changed the firing rate of spontaneously active MHb neurons. When blind patching from untagged MHb neurons in wild-type mice, we found only a subset (5/10 neurons) seemed to slow firing when the MOR agonist DAMGO (5 μM) was applied (Figures S1C–S1E). However, when recording from defined MOR^+^ neurons using MOR-Cre reporter mice (Figure 1B), DAMGO consistently and significantly inhibited MHb neuron firing, and this inhibition was reversed upon application of the MOR antagonist naloxone (Figures 1C–1E) (*p* = 0.0003, Friedman test). These data show that MOR is expressed in a subset of MHb neurons where it has inhibitory effects on cell firing consistent with canonical actions of MOR activation.

We next turned our attention to the IPN. Using both MOR-Cre reporter mice (Figure 1F) and RNAscope (Figure S1F), we found that a subset of IPN neurons expressed MOR. These cells were present across the rostral to caudal extent of IPN and showed notable density in IPR and intermediate IPN. To test how MOR activation influences IPN cells, whole-cell voltage-clamp recordings were made from untagged neurons in IPR (Figure 1G). We found that DAMGO application led to a steep increase in the holding current in a subset of recorded neurons (*n* = 12/23) (Figures 1H and 1I). This suggests that in a subset of IPN neurons, presumably those expressing MOR, MOR activation recruits a hyperpolarizing, or inhibitory, conductance. We also measured the rate of spontaneous excitatory postsynaptic currents (sEPSCs) in IPR neurons. The frequency of sEPSCs was also significantly reduced by DAMGO application (Figures 1J–1L) (*p* = 0.0002, Wilcoxon test), suggesting that spontaneous release from MOR-expressing excitatory inputs on to IPR neurons can be inhibited by MOR activation.

Overall, these data show that MOR is expressed in a subset of MHb and IPN neurons and that, at multiple locations within the HP circuit, MOR activation can drive canonical inhibitory effects.

### MOR activation potentiates evoked glutamate neurotransmission at the HP synapse

**Figure 2.**
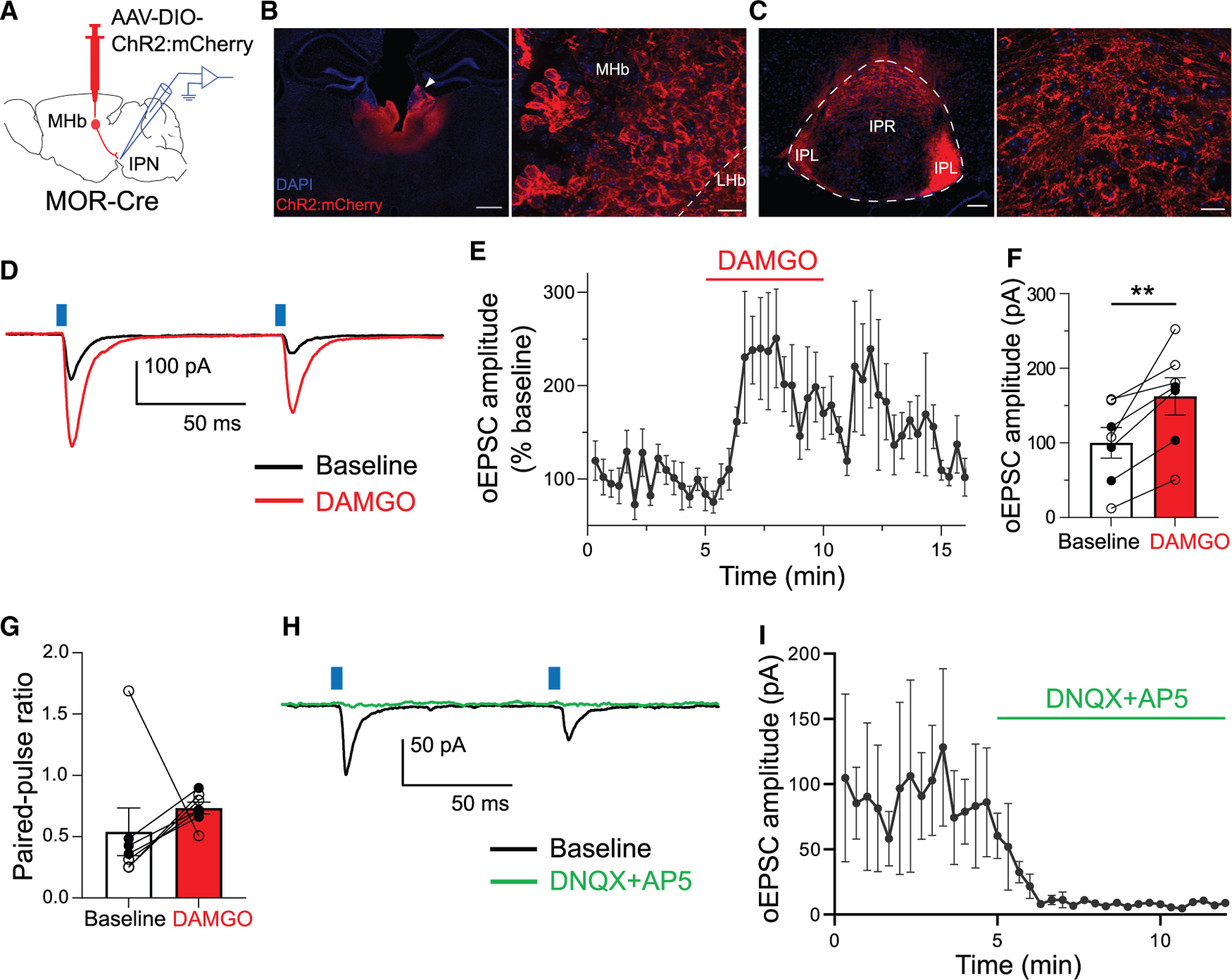
MOR activation enhances excitatory transmission at the HP synapse (A) Experiment design showing patch-clamp electrophysiology in the IPN following AAV injection into MHb of MOR-Cre mice. (B) (Left) The MHb injection site (white arrowhead). Scale bar, 500 μm. (Right) Magnified image showing ChR2:mCherry^+^ cells in MHb. Scale bar, 20 μm. (C) (Left) ChR2:mCherry^+^ projections in rostral (IPR) and lateral (IPL) IPN subregions. Scale bar, 100 μm. (Right) Magnified image of axonal fibers in IPR. Scale bar, 20 μm. (D) Traces from an example IPR neuron showing oEPSCs before and after the application of DAMGO (1 μM). (E and F) Averaged time trace (E) and bar graph (F) show DAMGO-mediated increase in oEPSC amplitude at the HP synapse (*n* = 7 cells/7 mice); paired t test, **p < 0.01. (G) Effects of DAMGO on PPR (*n* = 7 cells/7 mice); Wilcoxon test, *p* > 0.05. (H and I) Example trace (H) and averaged time trace (I) show that application of glutamate receptor blockers, DNQX (10 μM) plus AP5 (40 μM) abolished oEPSCs (*n* = 3 cells/3 mice). Solid and open circles displaying individual neurons (F and G) represent data from male and female mice, respectively. Data with error bars represent mean ± SEM.

Next, we investigated how MOR activation impacts evoked neurotransmission at the HP synapse. We injected AAV into the MHb of MOR-Cre mice (Figure 2A) to express ChR2: mCherry in MOR-expressing cells (Figure 2B) and their terminals in IPN, which were mainly observed in IPR and IPL subregions (Figure 2C). Spread of ChR2:mCherry expression was typically seen in MHb but also adjacent areas, including lateral habenula (LHb) and paraventricular thalamus (Figure 2B); however, these adjacent regions project little or none to the IPN.^13,27^ Following adeno-associated virus (AAV) injection, we made whole-cell patch-clamp (voltage-clamp) recordings from IPR neurons that were surrounded by ChR2:mCherry^+^ fibers. We delivered two pulses of blue light to assess fast EPSCs that were evoked in response to optogenetic stimulation (oEPSCs). We found that bath application of DAMGO consistently and significantly increased oEPSC amplitude (Figures 2D–2F) (t_6_ = 4.1; *p* = 0.006). Six of the seven cells showed paired-pulse depression that seemed to be attenuated by DAMGO (increase in paired-pulse ratio [PPR]), but in one cell this was the opposite, suggesting heterogeneity in this measure (Figure 2G) (*p* = 0.3, Wilcoxon test). In a subset of cells (*n* = 3), AMPA- and NMDA-type glutamate receptor blockers (DNQX and AP5) were applied after DAMGO washout and abolished the oEPSCs (Figures 2H and 2I), indicating that they were dependent on glutamate release.

We also tested the effects of MOR activation on evoked neurotransmission in IPN sections obtained from crossing MOR-Cre to Ai27 or Ai32 mice, which results in the Cre-dependent expression of ChR2:mCherry or ChR2:YFP in cells that expressed MORs. Similar to the AAV-based approach, we found that DAMGO led to an increase in oEPSC size in IPR neurons (Figures S2A–S2D) (t_4_ = 3.1; *p* = 0.035), and effects on PPR were again variable (Figure S2E) (*p* > 0.99; Wilcoxon test). However, in these experiments we observed low rates of connectivity, with only 8 of 41 patched IPR neurons showing oEPSCs. Moreover, in 17 of 41 IPR neurons we detected fast photocurrents with an onset latency of <1 ms, visible in the example trace (Figure S2B). These photocurrents are consistent with a subset of IPR neurons expressing MORs, but because of these complicating factors, we discontinued the use of this preparation.

Overall, our results indicate that MOR activation can result in a non-canonical potentiation of evoked glutamatergic transmission at the HP synapse.

### MOR potentiates excitatory neurotransmission at the HP synapse through a monosynaptic mechanism

**Figure 3.**
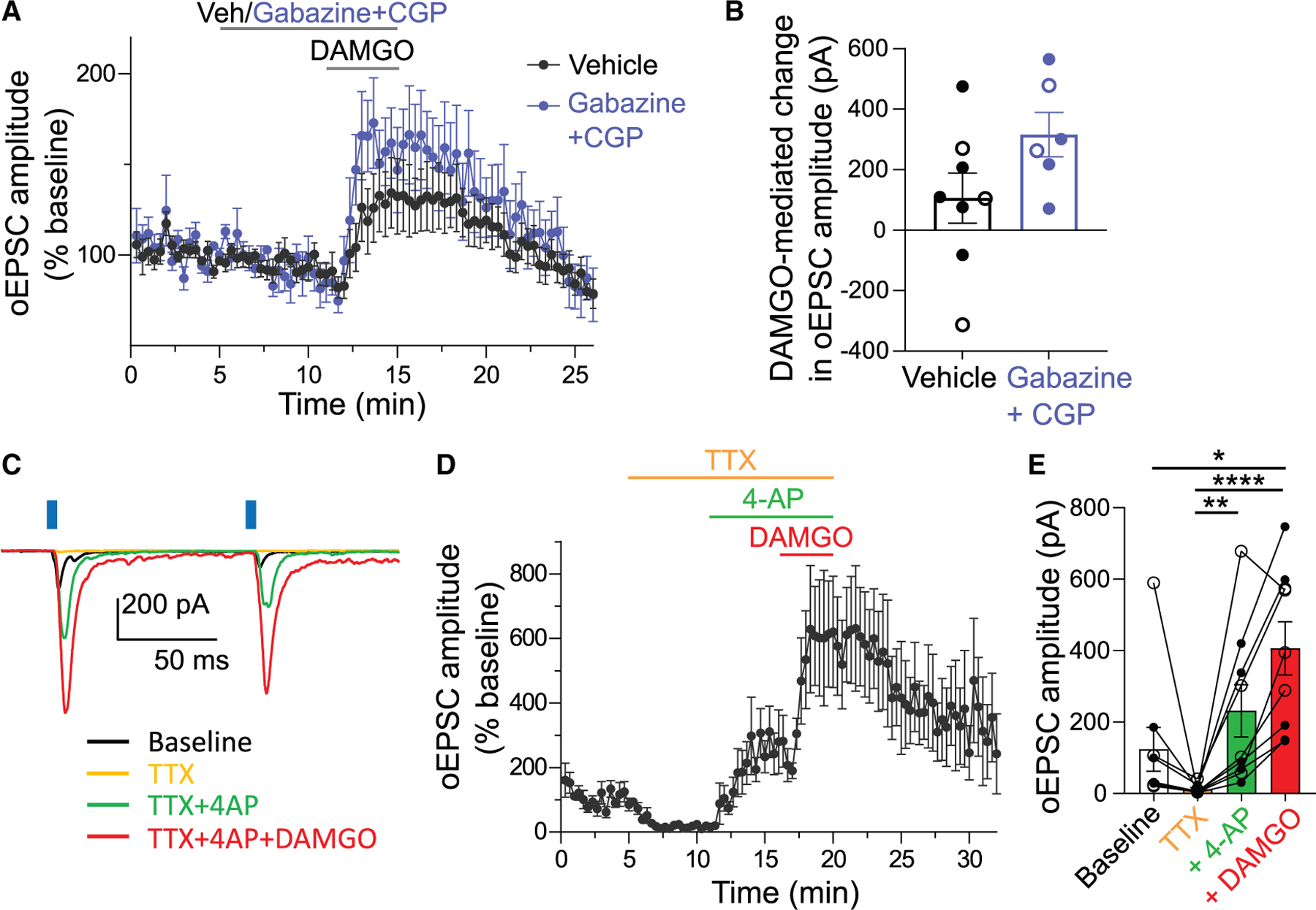
MOR-mediated potentiation of excitatory transmission at the HP synapse persists in the presence of GABA receptor blockers and TTX + 4-AP (A) Averaged time trace showing DAMGO (1 μM)-mediated increase in oEPSC amplitude at the HP synapse after pre-treatment with gabazine (5 μM) plus CGP (2 μM) (*n* = 6 cells/3 mice) or vehicle (0.04% DMSO in ACSF) (*n* = 8 cells/7 mice). (B) Effects of DAMGO on oEPSC amplitude persisted in the presence of gabazine plus CGP; unpaired t test, *p* > 0.05. (C–E) Example trace (C), averaged time trace (D, *n* = 6 cells/5 mice), and bar graph (E, *n* = 9 cells/8 mice) show DAMGO potentiation of oEPSC amplitude persisted in the presence of TTX + 4-AP. Friedman test, *p < 0.05, **p < 0.01, ****p < 0.0001. Solid and open circles displaying individual neurons (B and E) represent data from male and female mice, respectively. Data with error bars represent mean ± SEM.

The non-canonical potentiating effect of MOR activation on excitatory neurotransmission that we observed at the HP circuit was surprising; to our knowledge, such an effect has never been reported for MORs. We therefore began a series of studies to investigate the mechanism further. We first aimed to understand whether the oEPSC potentiation might be due to recruitment of a polysynaptic circuit. IPR neurons are primarily GABAergic and subsets of IPR neurons express MORs; thus, MOR activation on IPN neurons could lead to changes in GABA signaling that contribute to oEPSC potentiation. To test if the effects of MOR activation on excitatory neurotransmission at the HP synapse are dependent on indirect effects through local GABA signaling, we tested the effects of DAMGO in the presence of GABA_A_ and GABA_B_ receptor blockers gabazine and CGP-55845, at pharmacologically validated concentrations (Figures S3A and S5D). Following pre-treatment with either gabazine + CGP or vehicle, bath application of DAMGO increased the oEPSC amplitude at the HP synapse (Figures 3A and 3B) (main effect of treatment: F_1,12_ = 13.4, *p* = 0.003; group × treatment interaction: F_1,12_ = 3.3, *p* = 0.09). Notably, we observed a DAMGO-mediated inhibition in oEPSC amplitude in two neurons in this experiment (see below), both happened to be in the vehicle group (Figure 3B). Excitatory effects of DAMGO on neurotransmission were similarly observed in a separate group of neurons pre-treated with the GABA_A_ receptor blocker picrotoxin (Figures S3B and S3C) (main effect of treatment: F_1,5_ = 12.3, *p* = 0.02; group × treatment interaction: F_1,5_ = 0.01, *p* = 0.92). These results indicate that DAMGO-mediated potentiation of excitatory neurotransmission at the HP synapse persists in the presence of GABA receptor blockade and are therefore not dependent on recruitment of local GABA signaling mechanisms.

In addition to its facilitatory effects on evoked neurotransmission, MOR activation also inhibited spontaneous neurotransmission at the HP synapse (Figures 1J–1L and S4A). If the spontaneous co-release of glutamate and acetylcholine acting on metabotropic glutamate receptors (mGluRs) or muscarinic receptors resulted in a state of presynaptic depression, then MOR-mediated inhibition of spontaneous neurotransmission could relieve this presynaptic depression and augment release of glutamate. To test this hypothesis, we assessed the effects of DAMGO in the presence of the group 2 mGluR and muscarinic acetylcholine receptor blockers, LY-341495 and atropine. The DAMGO-mediated potentiation of oEPSC amplitude persisted after pre-treatment with atropine + LY-341495, which was reversed by naloxone application (Figures S3D–S3G). These results suggest that DAMGO’s facilitatory effects on evoked neurotransmission at the HP synapse are not mediated through the inhibition of spontaneous glutamatergic or cholinergic neurotransmission.

Next, to directly test if the potentiating effects of MOR activation on excitatory neurotransmission are monosynaptic, we first pharmacologically isolated the HP synapse by applying the voltage-gated sodium channel blocker tetrodotoxin (TTX) to prevent the propagation of action potentials, followed by the potassium channel blocker 4-aminopyridine (4-AP) to enhance the effects of optogenetic stimulation on MHb terminals. As before, oEPSCs were evoked in IPR cells under baseline conditions, followed by bath application of TTX, which effectively abolished these oEPSCs, then functional recovery of oEPSCs with the application of 4-AP. In these now monosynaptically isolated oEPSCs, we applied DAMGO and found that MOR activation again led to a significant potentiation of oEPSC amplitude (Figures 3C–3E) (*p* < 0.0001, Friedman test).

In this experiment, we noted that one cell showed an apparent DAMGO-mediated inhibition of neurotransmission (Figure 3E). In sum, and across the experiments presented, we found three cells that showed no clear DAMGO-mediated change in evoked neurotransmission and three cells that showed a DAMGO-mediated inhibition rather than potentiation of neurotransmission. We mapped the location of these cells and found that all three cells with inhibitory effects were located in caudal IPR (Figure S4B), suggesting that a subset of caudal IPR cells may not receive input from MOR-expressing MHb axons that show this non-canonical potentiation, but instead from a population of MOR-expressing axons that show canonical inhibitory effects on neurotransmission.

Overall, these results suggest that MOR activation at the HP synapse leads to net potentiation of glutamate transmission in a large majority of IPR cells through a monosynaptic mechanism.

### MOR activation potentiates excitatory transmission from HP cholinergic synapses

The potentiating effects of MOR activation on excitatory transmission are novel. However, activation of GABA_B_ receptors, another G_i_-coupled GPCR, were previously shown to potentiate excitatory neurotransmission from cholinergic terminals at HP synapses.^20^ We thus evaluated the effects of the GABA_B_ receptor agonist, baclofen, on neurotransmission at the HP synapse in MOR-Cre mice. Similar to DAMGO, baclofen significantly potentiated the oEPSC amplitude in IPR cells (Figures S5A–S5E) (t_6_ = 3.4; *p* = 0.01), with variable effects on PPR (Figure S5F) (t_6_ = 0.6; *p* = 0.56). These data suggest that MOR and GABA_B_ potentiate excitatory transmission through similar mechanisms from an overlapping population of HP synapses.

**Figure 4.**
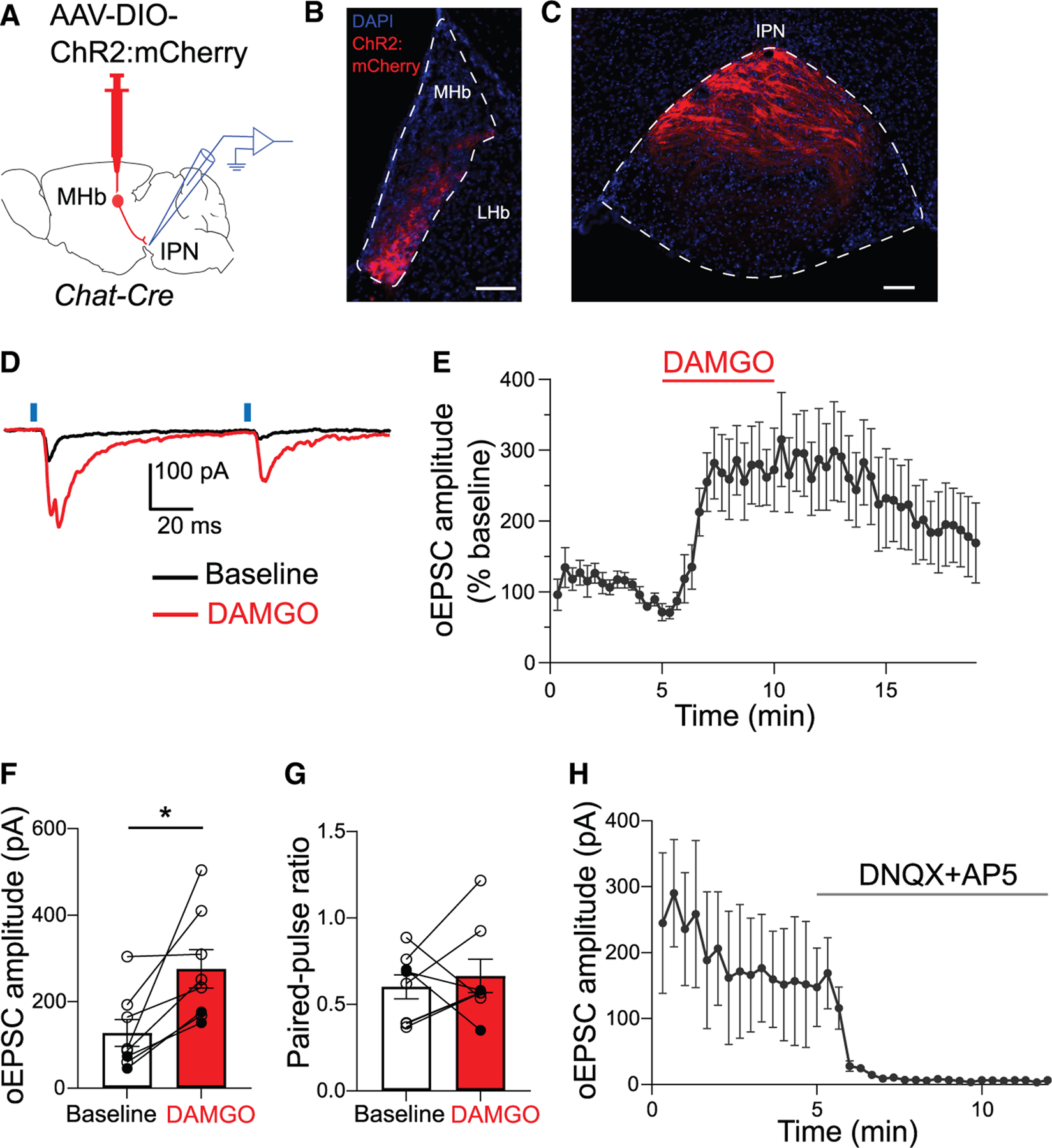
Glutamate co-release at cholinergic HP synapses is potentiated by MOR activation (A) Experiment design showing patch-clamp electrophysiology in IPN following AAV injection into MHb of *Chat-Cre* mice. (B and C) Images showing ChR2:mCherry expression in ventral MHb (B) and projections in IPR (C). Scale bars, 100 μm. (D–F) Example traces (D), averaged time trace (E), and bar graph (F) show DAMGO (1 μM) potentiation of oEPSC amplitudes at HP cholinergic synapses (*n* = 8 cells/6 mice); paired t test, *p < 0.05. (G) Effects of DAMGO on PPR (*n* = 8 cells/6 mice); Wilcoxon test, *p* > 0.05. (H) Application of glutamate receptor blockers, DNQX (10 μM) plus AP5 (40 μM) abolished oEPSCs in IPR neurons (*n* = 3 cells/3 mice). Solid and open circles displaying individual neurons (F and G) represent data from male and female mice, respectively. Data with error bars represent mean ± SEM.

Indeed, our RNAscope data (Figures S1A and S1B) demonstrate that a subset of cholinergic neurons located in ventrolateral MHb subareas express MOR. To test whether excitatory transmission from IPN-projecting cholinergic neurons is potentiated by MOR activation, we expressed ChR2: mCherry in MHb of *Chat-Cre* mice (Figure 4A). ChR2:mCherry expression was restricted to the ventral regions of MHb (Figure 4B) and observed throughout IPR (Figure 4C), a pattern consistent with cholinergic projections in the IPN.^9,20,28^ Using whole-cell patch clamp, we recorded fast oEPSCs in IPR that were significantly potentiated by DAMGO (Figures 4D–4F) (t_7_ = 3.3; *p* = 0.01). DAMGO’s effects on PPR were again variable (Figure 4G) (*p* = 0.64, Wilcoxon test). Following DAMGO washout, we applied glutamate blockers in a subset of cells (*n* = 3), which abolished the oEPSCs (Figure 4H), indicating they were dependent on glutamate release. Therefore, MHb cholinergic neurons co-release glutamate at the HP synapse, which is enhanced following MOR activation.

### DAMGO potentiates neurotransmission through presynaptic MORs at the HP synapse

**Figure 5.**
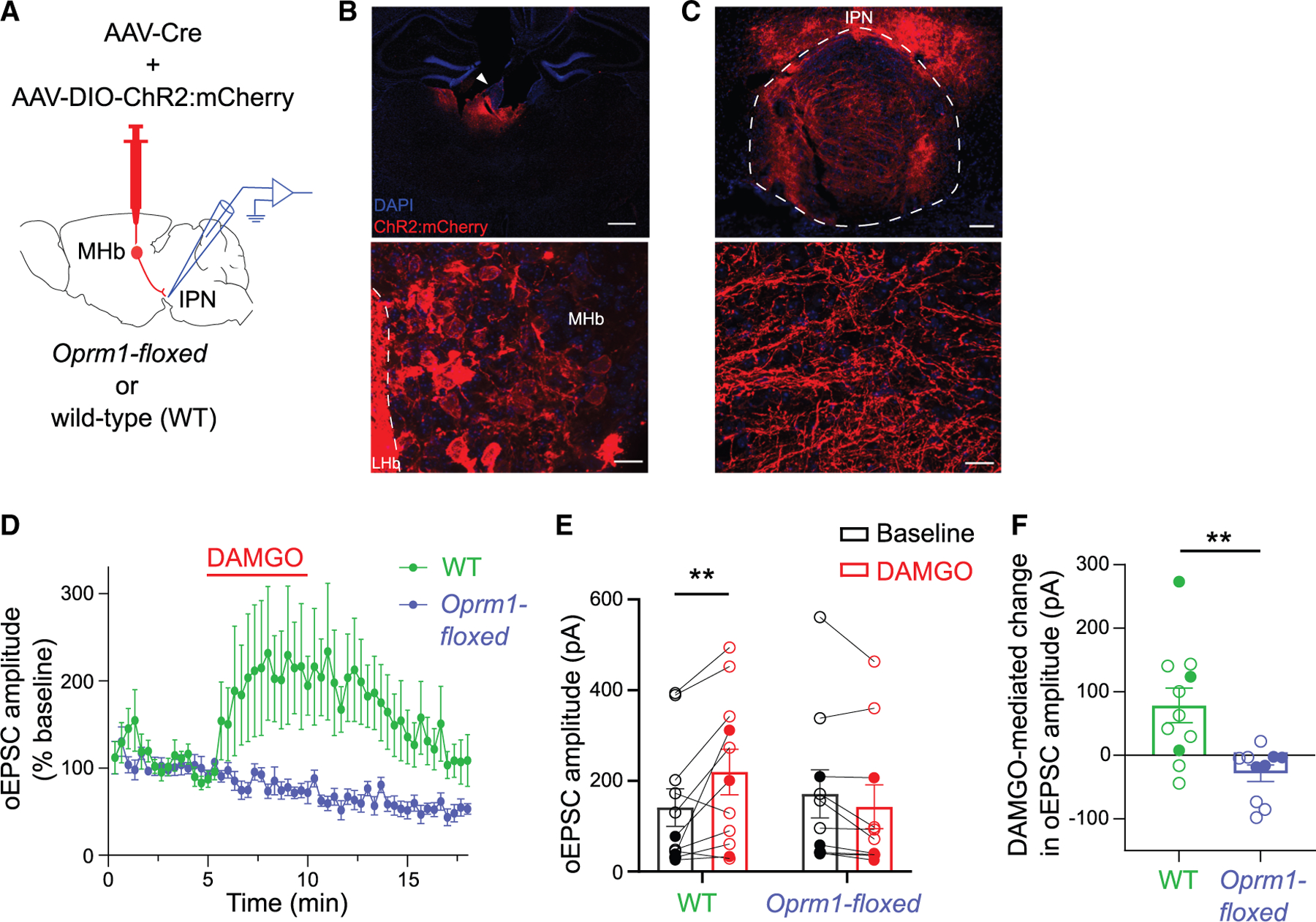
Pre-synaptic MOR is required for DAMGO-mediated potentiation of excitatory transmission at the HP synapse (A) Experimental design to delete MOR expression in presynaptic MHb neurons. (B) (Top) Expression of ChR2:mCherry following injection of Cre-recombinase (MHb, white arrowhead). Scale bar, 500 μm. (Bottom) Magnified image showing ChR2:mCherry^+^ cells in MHb. Scale bar, 20 μm. (C) (Top) ChR2:mCherry^+^ projections in the IPN. Scale bar, 100 μm. (Bottom) Magnified image of axonal fibers in IPR. Scale bar, 20 μm. (D and E) Averaged time trace (D) and bar graph (E) show DAMGO (1 μM) potentiation of oEPSC amplitude at the HP synapse in wild-type (WT) mice (*n* = 11 cells/8 mice), but not *Oprm1-floxed* mice (*n* = 10 cells/7 mice); mixed-model two-way ANOVA with Sidak post hoc multiple comparisons, **p < 0.01. (F) Significant potentiation of oEPSC amplitude by DAMGO in IPR neurons of WT compared to *Oprm1-floxed* mice; Mann-Whitney test, **p < 0.01. Solid and open circles displaying individual neurons (E and F) represent data from male and female mice, respectively. Data with error bars represent mean ± SEM.

The above data support the hypothesis that presynaptic MOR activation leads to the non-canonical potentiation of excitatory transmission at the HP synapse. To definitively test this hypothesis, we aimed to selectively disrupt MOR expression from pre-synaptic MHb neurons. To do so, we injected AAV-Cre into MHb of mice carrying a floxed *Oprm1* gene (*Oprm1*^*fl/fl*^). We also co-injected an AAV for Cre-dependent expression of ChR2:mCherry (Figure 5A). This approach will lead to the disruption of MORs and the simultaneous expression of ChR2:mCherry in neurons transduced by the AAVs, although ChR2 expression will not be restricted only to MOR-expressing neurons. Following injections, ChR2:mCherry expression was observed in MHb (Figure 5B) as well as terminals in IPN (Figure 5C). We waited >6 weeks to allow for MOR protein turnover before recording oEPSCs in IPR neurons. DAMGO-mediated potentiation of oEPSC amplitude was observed in wild-type control mice, but not in cells recorded from *Oprm1-floxed* mice (Figures 5D–5F) (group × treatment interaction: F_1,19_ = 11.7, *p* = 0.003; main effect of treatment: F_1,19_ = 2.6, *p* = 0.12). These results demonstrate that activation of presynaptic MOR is required for the non-canonical MOR-induced potentiation of excitatory transmission at the HP synapse.

## DISCUSSION

Activity in the HP circuit has been linked to the expression of avoidance and coping behaviors in response to aversive and anxiogenic stimuli, including in the context of addiction.^19,20,29–31^ Nicotinic acetylcholine receptors are densely expressed in the HP circuit and have been shown to contribute to aversive qualities of nicotine and nicotine dependence.^11,12,15,16,32^ Nicotine withdrawal increases the activity of IPN neurons, and dampening their excitability can alleviate nicotine-withdrawal symptoms.^33–35^ MORs are also densely expressed in the HP circuit.^5,6^ MOR-expressing neurons in the MHb contribute to the expression of affective and somatic features of opioid dependence.^21^ Furthermore, increased IPN activity was observed during opioid withdrawal, while ablation of descending IPN projection neurons reduced the aversive qualities of opioid withdrawal.^36^ Yet there had been almost nothing known about how MOR signaling influences activity of the HP circuit.

In this study, we used reporter and conditional knockout mice with optogenetics and patch-clamp electrophysiological recordings to establish both canonical and non-canonical effects of MOR activation on neurons and synapses in the HP circuit. MORs are G_i_-coupled GPCRs and their activation can increase potassium and inhibit calcium conductance, suppressing neuronal activity and neurotransmission.^25,26,37–39^ Consistent with these canonical mechanisms, we found that within the HP circuit MOR (1) inhibited firing in MOR-expressing MHb neurons, (2) activated an inhibitory hyperpolarizing conductance in IPN neurons, and (3) inhibited sEPSC frequency in IPN neurons.

In contrast with these canonical inhibitory effects, MOR activation markedly potentiated evoked glutamatergic transmission at the HP synapse. Through disinhibition, MOR activation can produce excitation in several brain regions including the hippocampus, periaqueductal gray matter, and ventral tegmental area.^40,41^ However, a direct facilitatory effect of MOR activation has not been reported for other synapses. IPN neurons are primarily GABAergic, and the activation of these neurons can regulate excitatory neurotransmission at the HP synapse through GABA release.^20,42^ However, the MOR-mediated facilitatory effect on glutamatergic transmission at the HP synapse persisted in the presence of GABA receptor blockade, indicating that this effect was not dependent on local GABA signaling. The facilitatory effect of MOR agonism also persisted when the optogenetic stimulation was monosynaptically isolated with TTX and 4-AP, demonstrating that it is not dependent on any other polysynaptic recruitment mechanism. Finally, genetic disruption of MORs in presynaptic MHb neurons completely abolished the facilitatory effect that we detected in postsynaptic IPN neurons. These results indicate that MOR activation on MHb axons has a non-canonical facilitatory effect on glutamate release.

What intracellular mechanisms downstream of MOR activation could potentiate evoked neurotransmitter release? Activation of GABA_B_ receptors, which are also G_i_-linked GPCRs, has been shown to similarly potentiate excitatory transmission from MHb cholinergic axons in IPN.^20,43^ Indeed, we observed a similar effect of GABA_B_ activation from MOR^+^ HP synapses, suggesting overlap in expression and GPCR signaling mechanisms between MOR and GABA_B_ at HP synapses. GABA_B_-mediated potentiation of glutamatergic transmission at the HP synapse was sensitive to pertussis toxin treatment and amplified presynaptic Ca^2+^ entry through Ca_v2.3_ channels^20^ but was not dependent on cAMP/PKA or PLC/PKC signaling pathways.^43^ MOR activation on MHb terminals could therefore be signaling through pertussis-toxin-sensitive G_i_-proteins, which non-canonically couple to the same set of Ca^2+^ channels as do the GABA_B_ receptors, thereby enhancing presynaptic Ca^2+^ influx and neurotransmitter release at the HP synapse.

An alternative possibility is that another signaling molecule, such as tonically released glutamate or acetylcholine, suppressed the evoked release of glutamate from the HP synapse at baseline and that MOR activation inhibits this suppression. To test whether such a mechanism might contribute, we repeated DAMGO application in the presence of blockers of metabotropic glutamate and muscarinic acetylcholine receptors; we found that DAMGO-mediated facilitation of evoked excitatory transmission persisted. It is therefore unlikely that spontaneously released glutamate or acetylcholine is leading to a basal state of synaptic depression at the HP synapse, yet we cannot rule out such a role for some other signaling molecule.

Although MOR activation potentiated evoked glutamatergic transmission in a large majority of cells across rostral to caudal IPR, an inhibitory effect was observed in a minority of IPR neurons. Each of these inhibitory responses were detected in caudal IPR neurons. One possibility is that some of the recorded neurons lie at the interface of caudal IPR and the rostral edge of median raphe, and these receive input from a different population of MOR-expressing neurons in either MHb or LHb.^17,27,44^ Indeed, MOR-Cre-expressing LHb neurons also expressed ChR2 in our preparation, and these cells project to the raphe and have been shown to express functional MORs.^45^

An alternative explanation relates to the observation that there are also multiple IPN-projecting cell types within the MHb.^8,46–49^ We show that MORs potentiate glutamate release from cholinergic neurons localizing to the ventral MHb but that MORs are prominently expressed in lateral and dorsal MHb populations that do not express cholinergic markers. Thus, MORs may potentiate excitatory transmission from cholinergic neurons, but inhibit excitatory transmission at some or all of the non-cholinergic HP synapses.^50^ In that event, we may be measuring the net effects of evoked glutamate release from a mix of HP synapses that are canonically inhibited and non-canonically potentiated, although the potentiating effects of MORs are clearly more prominent in our preparation.

Yet in the same postsynaptic IPR cells where we observed pronounced MOR-induced potentiation of evoked excitatory transmission, we detected MOR-induced inhibition in the rate of spontaneous excitatory events. This may indicate that MOR activation simultaneously suppresses background spontaneous neurotransmission while potentiating evoked neurotransmission from habenular inputs onto IPN cells. Interestingly, the activation of GABA_B_ receptors shows the same pattern of differential effects on evoked and spontaneous transmission at the HP synapse.^43^ These phenomena could arise due to differential GPCR modulation of evoked and spontaneous release arising from distinct pools of vesicles.

Repeated exposure to exogenous opioids can lead to receptor desensitization and tolerance that reduces the number of functional receptors.^51,52^ Opioid-induced changes in MOR signaling within the HP circuit have not been assessed, but MORs in the HP circuit can contribute to the manifestation of behaviors linked to opioid dependence and withdrawal.^21,36^ Future work must therefore investigate how chronic exposure to exogenous opioids changes the HP circuit, and it will be important to assess how MOR signaling changes within distinct cellular and subcellular compartments.

In this study, we report the physiological consequences of MOR activation in the HP circuit. Canonical inhibitory effects were observed in the somatodendritic compartments of MHb and IPN neurons. However, we also revealed a novel facilitatory effect of MOR on evoked neurotransmission at the HP synapse. These results point to a new mode by which MORs can signal in presynaptic terminals and provide a new target through which opioids may act to induce neuroadaptations that underlie opioid addiction.

### Limitations of the study

Perhaps the main limitation of our study is the lack of data explaining how intracellular MOR signaling can drive non-canonical facilitatory effects on excitatory transmission at the HP synapse. In other synapses, the effects of MOR signaling have been inhibitory. Indeed, even in IPN neurons in which evoked excitatory transmission is facilitated by MOR agonism, spontaneous excitatory transmission is inhibited. Solving this puzzle will be important to fully establish the non-canonical effect of MOR at the HP synapse, although we note that important progress has been made in recent studies on the facilitatory actions of GABA_B_ at the HP synapse, as discussed above, and it is reasonable to suppose similar mechanisms may apply to MOR actions at the HP synapse. Another limitation is that we focused only on glutamate release at the HP synapse. However, subsets of MHb neurons also release the neurotransmitter acetylcholine, as well as neuropeptides such as the tachykinins. Our study leaves open many other questions, including how the canonical and non-canonical effects of MOR signaling shape the overall response of HP circuit outputs to exogenous and endogenous opioid signaling, what is the source of endogenous opioids that activate MORs throughout the HP circuit, how opioid actions on the HP circuit shape behavior, and how chronic opioid intake or opioid addiction change the HP circuit.

In December 2024, a pre-print version of this article^53^ was submitted in coordination with a similar study from the McBain laboratory (National Institutes of Health).^50^

## RESOURCE AVAILABILITY

### Lead contact

Requests for further information, resources, and reagents should be directed to the lead contact, Thomas S. Hnasko (thnasko@health.ucsd.edu).

### Materials availability

This study did not generate new unique reagents.

### Data and code availability

- Source data have been deposited at Zenodo and are publicly available as of the date of publication at https://doi.org/10.5281/zenodo.15671305.
- Requests for any additional information that may be required to reanalyze data reported in this paper can be made to the lead contact.
- This paper does not make use of original code.

## STAR★METHODS

### EXPERIMENTAL MODEL AND STUDY PARTICIPANT DETAILS

Mice were bred at University of California San Diego (UCSD) and group housed on a 12-h light/dark cycle with *ad libitum* access to pelleted food and water. Initial breeders wild-type (C57Bl/6J, Stock: 000664), Ai6 ZsGreen reporter (Stock: 007906), Ai14 tdTomato reporter (Stock: 007914), Ai27D ChR2:tdTomato reporter (Stock: 012567), Ai32 ChR2:eYFP reporter (Stock: 024109), *Chat*^*Cre/Cre*^ (Stock: 006410) and *Oprm1*^*fl/fl*^ (Stock: 030074) were obtained from Jackson laboratories. *Oprm1* (MOR)-Cre mice were initially obtained from Dr. Brigitte Kieffer (McGill University) and maintained back-crossed on to C57BL/6J.^54^ Male and female mice were used for all experiments in approximately equal proportion as mice became available. All experiments were performed in accordance with protocols approved by UCSD Institutional Animal Care and Use Committee.

### METHOD DETAILS

#### Stereotactic surgeries

For intracranial injections, mice (5–8 weeks) were deeply anesthetized with isoflurane and placed in a stereotaxic apparatus (Kopf Instruments). For optogenetic experiments testing effects of DAMGO in MOR-Cre or *Chat-Cre* mice, bilateral injections (150 nL/hemisphere) of AAV5-EF1a-DIO-ChR2:mCherry (2–2.5 × 10^13^ gc/ml, Addgene 20297) were made into MHb. For optogenetic experiments testing effects of baclofen in MOR-Cre, bilateral injections (150 nL/hemisphere) of AAV5-EF1a-DIO-ChR2:eYFP (1.8 × 10^13^ gc/ml, Addgene 20298) were made into MHb. For experiments testing effects of DAMGO in *Oprm1*^*fl/fl*^ (*Oprm1-floxed*) or wild-type (WT) mice, AAV5-hSyn-Cre (Addgene 105553) was combined with AAV5-Ef1a-DIO-ChR2:mCherry. Bilateral injections (150 nL/hemisphere) of this mixture were made into MHb at final concentrations of 1.4–5.3 × 10^12^ gc/ml (AAV-Cre) and 1.9 × 10^13^ gc/ml (AAV-DIO-ChR2:mCherry). Injections into MHb were made at a 20° medio-lateral angle at coordinates (mm relative to bregma): ML = ±1.09, AP = −1.58, DV = −2.56. Injections were made using pulled glass pipettes (∼25 μm aperture diameter) and a Nanoject 3 (Drummond Scientific) at a rate of 10 nL/s with 1-s pulse and 5-s inter-pulse interval. The glass pipettes were held in place for 5 min after infusion before retracting. Animals were treated with a topical antibiotic and administered with the analgesic carprofen (5 mg/kg s.c.; Rimadyl). MOR-Cre or *Chat-Cre* mice were allowed to recover for >3 weeks, whereas *Oprm1-floxed* and WT mice were allowed to recover for >6 weeks before proceeding.

#### Patch-clamp electrophysiology

Mice were anesthetized on the day of experiments with an intraperitoneal (IP) injection of sodium pentobarbital (200 mg/kg, Virbac) and transcardially perfused with 15–20 mL of ice-cold oxygenated N-methyl D-glucamine (NMDG)-artificial cerebrospinal fluid (ACSF) containing (in mM): 92 NMDG, 2.5 KCl, 1.25 NaH_2_PO_4_, 30 NaHCO_3_, 20 HEPES, 25 D-glucose, 2 thiourea, 5 Na-ascorbate, 3 Na-pyruvate, 0.5 CaCl_2_ and 10 MgSO_4_. Animals were decapitated and brains rapidly extracted then submerged in ice-cold NMDG-ACSF. 200-μm coronal brain slices containing the IPN or MHb were obtained using a Leica VT 1200S vibratome and transferred to a recovery chamber containing 150 mL of preheated (32°C–34°C), oxygenated NMDG-ACSF. A 2 M Na+ spike-in solution was then added to the recovery chamber in increasing volumes and 5-min increments to achieve a controlled rate of Na+ reintroduction,^55^ following which slices were transferred to oxygenated ACSF at room temperature containing (in mM): 115 NaCl, 2.5 KCl, 1.23 NaH_2_PO_4_, 2 MgSO_4_, 10 D-glucose, 2 CaCl_2_, 26 NaHCO_3_, 2 thiourea, 5 Na-ascorbate, and 3 Na-pyruvate. Slices were allowed to equilibrate for at least one hour before recordings, following which they were transferred to a recording chamber maintained at ∼31°C and perfused continuously at a rate of 1.5–2 mL/min with oxygenated ACSF that contained (in mM): 125 NaCl, 2.5 KCl, 1.2 NaH_2_PO_4_, 2 MgSO_4_, 12.5 D-glucose, 2 CaCl_2_, and 26 NaHCO_3_. All solutions were saturated with carbogen (95% O_2_ + 5% CO_2_) to maintain a pH of ∼7.3.

For cell attached recordings, brains were extracted from either MOR-Cre x *Rosa26*-tdTomato reporter mice (4–5 weeks) or wild-type (c57) mice (4–6 weeks). Using reporter mice, MOR^+^ neurons in MHb were visualized through epifluorescence imaging with a 40× water-immersion objective and a Zeiss filter set, and visually guided patch-clamp recordings were made using infrared-differential interference contrast (IR-DIC) illumination (Axiocam MRm, Examiner.A1, Zeiss). Wild-type mice were used to make patch-clamp recordings from untagged neurons in lateral MHb. Cell-attached recordings were made using 5–7 MΩ patch pipettes filled with a K^+^ gluconate-based internal solution containing (in mM): 130 K-gluconate, 2 KCl, 2 MgCl_2_, 2 Na_2_-ATP, 0.3 Na-GTP, 10 phosphocreatine, 10 HEPES and 0.2 EGTA. Patch pipettes were prepared from thin walled (1.5 mm/1.1 mm) borosilicate glass capillaries (Sutter Instruments) using a micro-pipette puller (Narishige, PC-10). Cell-attached recordings were made in voltage-clamp mode with command voltage set so holding current was approximately zero. Baseline firing was recorded for 5 min followed by 5 min of DAMGO (5 μM) application then 5 min of DAMGO (5 μM) + Naloxone (5 μM) application. The firing frequency was calculated as the average of the last 3 min of each condition.

For whole-cell recordings and optogenetic experiments, brain slices were obtained from adult mice (8–15 weeks). Using IR-DIC, patch-clamp recordings were made from IPR cells adjacent to mCherry- or eYFP-positive fibers which were visualized with epifluorescence imaging using a 40× water-immersion objective and a Zeiss filter set. Whole-cell voltage-clamp recordings were made with patch pipettes pulled to an open tip resistance of 6–7 MΩ and filled with either a K-gluconate internal solution (as described above) or a cesium-based internal solution containing (in mM): 130 CsMeSO_3_, 3 MgCl_2_, 4 Na_2_-ATP, 0.25 Na-GTP, 10 phosphocreatine, 10 HEPES, 0.2 EGTA, and 5 QX-314. Internal solutions were adjusted to a pH of ∼7.3 and ∼285mOsm and used consistently within an experiment. To record optogenetic-induced excitatory postsynaptic currents (oEPSCs), cells were voltage-clamped at −65 mV and two pulses of blue light (2 or 5 ms) were flashed at 10 Hz through the light path of the microscope using a light-emitting diode (UHP-LED460, Prizmatix) under computer control to activate ChR2. The paired light pulses were delivered every 20 s. In all experiments, oEPSCs were recorded for a baseline period of 5 min, DAMGO (1 μM) was bath applied for either 4 or 5 min, followed by washout. Light-pulse width and DAMGO application duration was kept consistent within an experiment. Changes in amplitude of the first oEPSC were averaged and reported in graphs. Baseline oEPSC amplitude was averaged from the last 3 min of baseline recording; the oEPSC amplitude in response to DAMGO was averaged from the last 2 or 3 min of 4- or 5-min DAMGO application. Glutamate receptor blockers DNQX (10 μM) and AP5 (40 μM) were bath applied ∼10 min after DAMGO washout to test effects of glutamate receptor blockade on oEPSCs. For experiments testing effects of DAMGO with Gabazine and CGP, after baseline recording of oEPSCs, Gabazine (5 μM) and CGP-55845 (2 μM) was bath applied for 6 min followed by application of DAMGO + Gabazine + CGP for 4 min. For control experiments, vehicle (0.04% DMSO in ACSF) was applied instead of Gabazine+CGP. For experiments testing effects of DAMGO with picrotoxin (PTX) and CGP, after baseline recording of oEPSCs, PTX (50 μM) and CGP (2 μM) was bath applied for 10 min followed by application of DAMGO + PTX + CGP for 5 min. For control experiments, vehicle (0.09% DMSO in ACSF) was applied instead of PTX+CGP. For experiments testing effects of DAMGO with tetrodotoxin (TTX) and 4-aminopyridine (4-AP), after baseline recording of oEPSCs, TTX (1 μM) was bath applied for 6 min followed by application of 4-AP (100 μM) + TTX for 5 min, followed by application of DAMGO + 4-AP + TTX for 4 min. The oEPSC amplitude in response to TTX was averaged from the last 3 min of its application before 4-AP, and the oEPSC amplitude in response to 4-AP was averaged from the last 2 min of its application before DAMGO. TTX and 4-AP application duration was different in 3 out of 9 cells and therefore not included in the averaged time-trace graph. For experiments testing effects of DAMGO with atropine and LY-341495, after baseline recording of oEPSCs, atropine (10 μM) and LY-341495 (1 μM) was bath applied for 6 min followed by application of DAMGO + atropine + LY-341495 for 5 min. In 3/10 cells, naloxone (5 μM) was applied after DAMGO for 5 min. The oEPSC amplitude in response to atropine + LY-341495 was averaged from the last 3 min of its application before DAMGO. For testing the effects of baclofen on oEPSC amplitude, baclofen (5 μM) was bath applied for 5 min after baseline recording and the oEPSC amplitude was averaged from the last 3 min of its application. The paired-pulse ratio (PPR) was calculated by dividing the mean oEPSC amplitude in response to the second pulse of optogenetic stimulation by the mean oEPSC amplitude in response to the first pulse of optogenetic stimulation (oEPSC2/oEPSC1). oEPSC2 and oEPSC1 were averaged from the last 3 min of 5-min baseline recording or DAMGO or baclofen application. The holding current was extracted from the last 10 s of the 20 s sweep from cells which were patched with the K-gluconate internal solution. Cells with a rate of change of holding current (dI/dt) > 10 pA/min after DAMGO application were categorized as responding cells. Changes in the frequency of spontaneous excitatory postsynaptic currents (sEPSCs) were also monitored in a subset of these cells in response to DAMGO application. Baseline and DAMGO holding current was averaged from the last 3 min of baseline recording and DAMGO application, respectively. Baseline sEPSC frequency was averaged from the last 3 min of baseline recording, whereas during DAMGO, was averaged from a 2-min window, one minute after start of DAMGO application. Experiments and data analysis for testing effects of DAMGO on oEPSCs in WT or *Oprm1-floxed* mice were performed by experimenter blind to genotype. Access resistance (Ra) was calculated from a 10-mV hyperpolarizing pulse applied at the beginning of each sweep and monitored for whole-cell recordings. Cells with a Ra change> 30% were excluded from analyses.

Recordings were made using a Multiclamp 700B Amplifier (Axon Instruments), lowpass filtered at 2 kHz, digitized at 20 kHz (Axon Digidata 1550A, Axon Instruments), collected and analyzed using Clampex and Clampfit v10 software (Molecular Devices). All drugs were added to the ACSF following dilution of a stock solution stored at −20°C. All drugs were purchased from Tocris, except PTX and atropine, which were purchased from Sigma-Aldrich.

#### RNAscope

Adult female wild-type mice (10 and 19 weeks) were anesthetized with an IP injection of sodium pentobarbital (200 mg/kg) and transcardially perfused with 20 mL UltraPure water-based 1x PBS (water from Invitrogen; 10x PBS from Fisher Bioreagents) followed by 20 mL 4% PFA at a rate of 6 mL/min. Brains were extracted, fixed in 4% PFA overnight in 4°C and transferred to a 30% sucrose solution (in PBS) for 24h at 4°C. 20-μm-thick coronal sections containing MHb or IPN were made using a Leica CM3050S cryostat and slices were collected in PBS and mounted on Superfrost Plus microscope slides (Fisher Scientific). RNAscope *in-situ* fluorescent hybridization was done as previously published^56^ using probes (Advanced Cell Diagnostics) against *Oprm1*, *Chat,* and *Slc17a6* (VGLUT2). Four mice were sacrificed to obtain MHb or IPN sections, which were imaged with an Axio Observer.Z1 fluorescent microscope using ApoTome.2 system and Plan-APOCHROMAT 20x/0.8 objective (Zeiss).

#### Histology and fluorescent imaging

Native fluorescence was imaged from sections expressing ZsGreen or mCherry in *Chat-Cre* mice. The mCherry signal in MOR-Cre, WT and *Oprm1-floxed* mice was immunoamplified. Mice were anesthetized with an IP injection of sodium pentobarbital (200 mg/kg; i. p.) and transcardially perfused with 20 mL of phosphate-buffered saline (PBS) followed by 20 mL 4% paraformaldehyde (PFA) at a rate of 6 mL/min. Brains were extracted, post-fixed in 4% PFA at 4°C overnight, and transferred to 30% sucrose in PBS for 48 h at 4°C. Brains were flash frozen in isopentane and stored at −80°C. 30-μm coronal sections containing the MHb and IPN were obtained using a cryostat (CM3050S, Leica) and collected in PBS containing 0.01% sodium azide. For immunostaining, brain sections were gently rocked 3 × 5 min in PBS, 3 × 5 min in PBS containing 0.2% Triton X-100 (PBST) and blocked with 4% normal donkey serum (NDS) in PBST for 1 h at room temperature (RT). Sections were then incubated in primary antibody rabbit anti-DsRed (1:2000; Takara #632496) or chicken anti-RFP (1:1000; Rockland # 600–901-379) added to 4% NDS block solution at 4°C overnight. Following this, sections were rinsed 3 × 10 min with PBST and incubated in secondary antibody Alexa 594 donkey anti-rabbit (1:400; Jackson ImmunoResearch) or Alexa 594 donkey anti-chicken (1:400; Jackson ImmunoResearch) for 2 h at RT. Sections were washed 3 × 10 min with PBS, mounted on slides, and coverslipped with Fluoromount-G mounting medium (Southern Biotech) containing DAPI (0.5 μg/mL; Roche). Images were acquired using widefield epifluorescence (Zeiss AxioObserver) with a 10X and 63× objective using ApoTome.2 system. MHb subnuclei borders were drawn based on Juárez-Leal et al.^57^ IPN subnuclei borders were drawn based on ‘‘The Mouse Brain in Stereotaxic Coordinates’’ (Paxinos, G. and Franklin, K.B.J. (2001) The Mouse Brain in Stereotaxic Coordinates. 2nd Edition, Academic Press).

### QUANTIFICATION AND STATISTICAL ANALYSIS

Statistical analysis was performed using GraphPad Prism v10. All data are represented as mean ± standard error of the mean (SEM) and/or as individual points. Data was tested for normality using the Shapiro-Wilk test and parametric or non-parametric tests were used as appropriate. Data was analyzed using paired t test or Wilcoxon test, unpaired t test or Mann-Whitney test, repeated-measures one-way ANOVA or Friedman test and mixed-model two-way ANOVA followed by Sidak post hoc multiple comparisons. Significance was set at *p* < 0.05. Details of statistical tests used and sample sizes can be found in results and in figure legends.

## Supplementary Material

1

Supplemental information can be found online at https://doi.org/10.1016/j.celrep.2025.115874.

## Figures and Tables

**Table:** KEY RESOURCES TABLE

REAGENT or RESOURCE	SOURCE	IDENTIFIER
Antibodies

Rabbit anti-DsRed	Takara Bio	Cat# 632496; RRID: AB_10013483
Alexa Fluor 594 AffiniPure Donkey Anti-Rabbit IgG	Jackson ImmunoResearch	Cat# 711–585-152; RRID: AB_2340621
Chicken anti-RFP	Rockland	Cat# 600–901-379; RRID: AB_10704808
Alexa Fluor 594 AffiniPure Donkey Anti-Chicken IgG	Jackson ImmunoResearch	Cat# 703–585-155; RRID: AB_2340377

Bacterial and virus strains		

AAV5-EF1a-DIO-hChR2(H134R)-mCherry	Addgene	RRID: Addgene_20297
AAV5-EF1a-DIO-hChR2(H134R)-EYFP	Addgene	RRID: Addgene_20298
AAV5-hSyn-Cre	Addgene	RRID: Addgene_105553

Chemicals, peptides, and recombinant proteins		

DAMGO	Tocris	Cat# 1171
Naloxone hydrochloride	Tocris	Cat# 0599
DNQX	Tocris	Cat# 2312
DL-AP5	Tocris	Cat# 3693
Gabazine	Tocris	Cat# 1262
CGP-55845 hydrochloride	Tocris	Cat# 1248
Tetrodotoxin (TTX)	Tocris	Cat# 1069
4-aminopyridine (4-AP)	Tocris	Cat# 0940
Picrotoxin (PTX)	Sigma-Aldrich	Cat# P1675
Baclofen	Tocris	Cat# 0796
Atropine	Sigma-Aldrich	Cat# PHR3846
LY-341495	Tocris	Cat# 1209
QX-314 bromide	Tocris	Cat# 1014

Experimental models: Organisms/strains

Mouse: *Oprm1* (MOR)^*Cre/Cre*^: C57BL/6-*Oprm1*^*tm2.1(EGFP/cre)Ics*^/KffJ	Bailly et al.^54^	RRID:IMSR_JAX:038053
Mouse: Wild-type (WT): C57BL/6J	The Jackson Laboratory	RRID:IMSR_JAX:000664
Mouse: Ai6: B6.Cg-*Gt(ROSA) 26Sor*^*tm6(CAG-ZsGreen1)Hze*^/J	The Jackson Laboratory	RRID:IMSR_JAX:007906
Mouse: Ai14: B6.Cg-*Gt(ROSA) 26Sor*^*tm14(CAG-tdTomato)Hze*^/J	The Jackson Laboratory	RRID:IMSR_JAX:007914
Mouse: Ai27D: B6.Cg-*Gt(ROSA) 26Sor*^tm27.1(CAG-COP4*H134R/tdTomato)Hze^/J	The Jackson Laboratory	RRID:IMSR_JAX:012567
Mouse: Ai32: B6.Cg-*Gt(ROSA) 26Sor*^tm32(CAG-COP4*H134R/EYFP)Hze^/J	The Jackson Laboratory	RRID:IMSR_JAX:024109
Mouse: *Chat*^*Cre/Cre*^: B6;129S6-*Chat*^*tm2(cre)Lowl*^/J	The Jackson Laboratory	RRID:IMSR_JAX:006410
Mouse: *Oprm1*^*fl/fl*^: B6;129-*Oprm1*^*tm1.1Cgrf*^/KffJ	The Jackson Laboratory	RRID:IMSR_JAX:030074

Software and algorithms		

pClamp software v10	Molecular Devices	RRID:SCR_011323
Zen 2 Blue	Zeiss	RRID:SCR_013672
GraphPad Prism v10	GraphPad Software	RRID:SCR_002798
Inkscape	Inkscape	RRID:SCR_014479

Deposited data		

Electrophysiology data	This paper	Zenodo: https://doi.org/10.5281/zenodo.15671305

Other		

Nanoject III	Drummond Scientific	Cat# 3–000-207
Small Animal Stereotaxic Instrument with Digital Display Console, Model 940	David Kopf Instruments	N/A
Leica CM3050S Cryostat	Leica	RRID:SCR_020206
Leica VT1200S vibratome	Leica	RRID:SCR_018453
Pipette puller PC-10	Narishige	RRID:SCR_022057
Axio Examiner A1 microscope	Zeiss	RRID:SCR_025040
Axio Observer Z1 microscope	Zeiss	RRID:SCR_021351
Multiclamp 700B Amplifier	Axon instruments	RRID:SCR_018455
Axon Digidata 1550A	Axon instruments	N/A

## Highlights

- MOR is expressed in MHb and IPN neurons, where it is canonically inhibitory
- MOR facilitates glutamatergic transmission at cholinergic HP synapses
- The facilitatory effect of MOR depends on a monosynaptic mechanism
- Pre-synaptic MOR non-canonically facilitates excitatory transmission at HP synapses
