# Supplementary material for: Mu-opioid receptor activation potentiates excitatory transmission at the habenulo-peduncular synapse
Source: Cell Rep. Author manuscript; Available in PMC 2025 Aug 14. (PMC12352575; doi:10.1016/j.celrep.2025.115874)
Supplement: 1 [file NIHMS2099500-supplement-1.pdf]

Cell Reports, Volume *44*

Supplemental information

Mu-opioid receptor activation potentiates excitatory transmission at the habenulo-peduncular synapse

Sarthak M. Singhal, Agata Szlaga, Yen-Chu Chen, William S. Conrad, and Thomas S. Hnasko

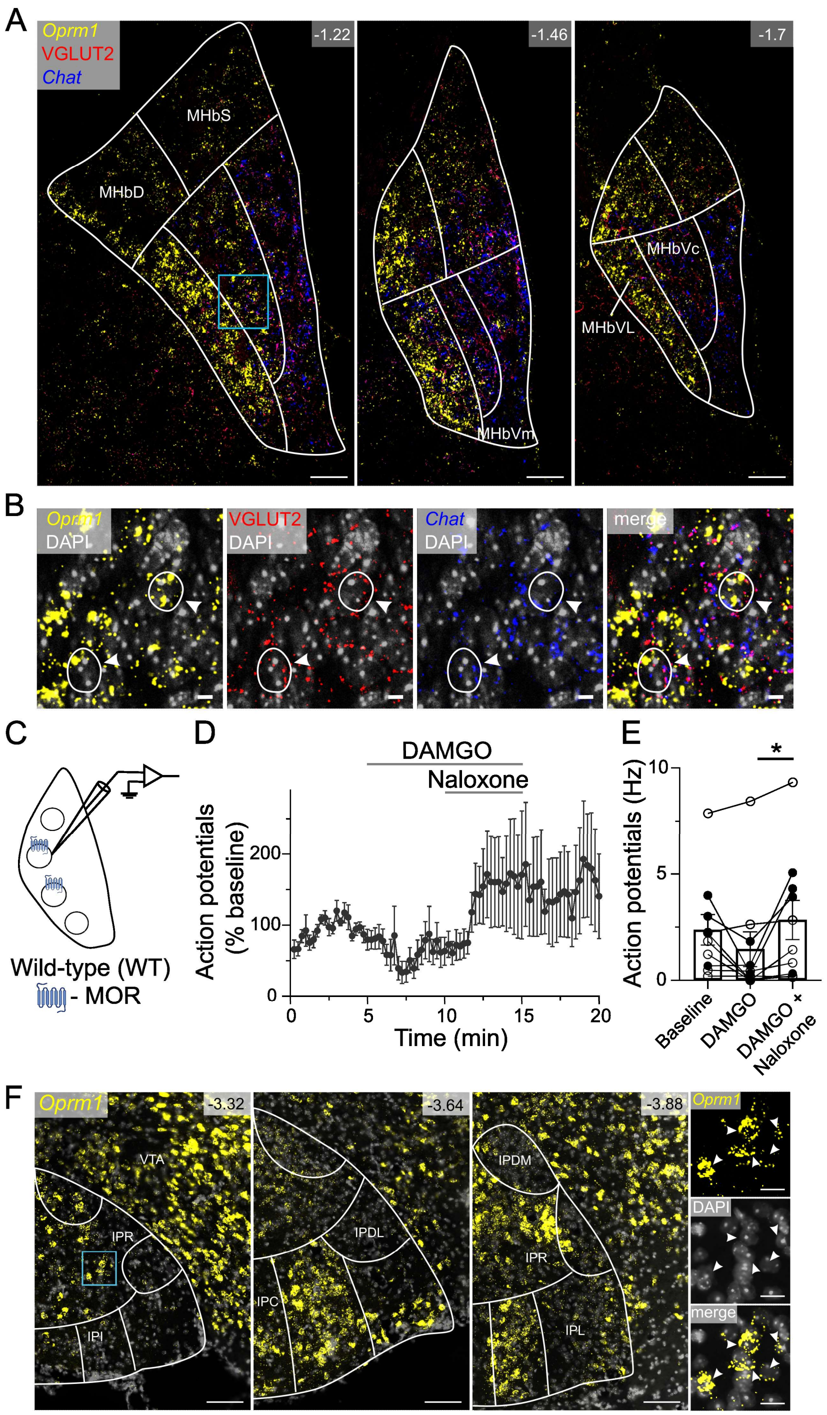

Figure S1 (related to Figure 1). MOR expression in MHb and IPN

(A) MHb labeled using probes targeting *Oprm1* (MOR, yellow), *Chat* (blue), and *Slc17a6* (VGLUT2, red); coordinates noted relative to bregma (in mm); scale, 50 µm.

(B) Magnified image from inset in panel A (marked by blue square). Arrowheads and outlines indicate MHb neurons co-expressing *Oprm1*, *Chat* and VGLUT2; scale, 20 µm.

(C) Schematic of cell-attached recordings from unlabeled/unidentified lateral MHb cells, some of which express MORs.

(D and E) Averaged time-trace (D) and bar-graph (E) of cell-attached recordings showing effects of DAMGO (5 μM) and naloxone (5 μM) on action-potential firing in MHb neurons (n= 10 cells/ 4 mice); Friedman test, *$p<0.05$. DAMGO application inhibited firing in a subset of neurons (n= 5/10), reversed by naloxone, consistent with MOR expression in a subset of MHb neurons. Solid and open circles showing individual neurons represent data from male and female mice, respectively.
(F) IPN labeled using probes targeting *Oprm1* (MOR, yellow); coordinates noted relative to bregma (in mm); scale, 100 μm. Right panel set shows magnified area within blue square; arrowheads indicate IPN neurons expressing *Oprm1*; scale, 20 μm.

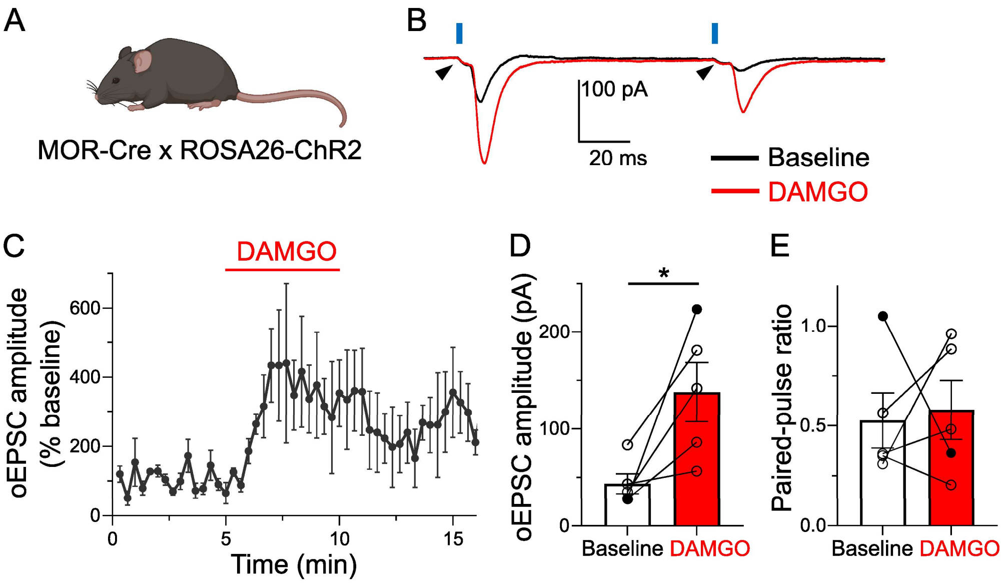

Figure S2 (related to Figure 2). MOR activation potentiates excitatory neurotransmission in the IPR
(A) MOR-Cre crossed to Ai27 or Ai32 mice resulting in Cre-dependent expression of ChR2:mCherry or ChR2:YFP in cells that expressed MOR.
(B) Example traces from IPR neuron showing oEPSCs before and after DAMGO (1 μM) application. Black arrowhead indicates photocurrent, suggestive of a MOR^{+} postsynaptic IPR neuron expressing ChR2.
(C and D) Average time trace (C) and bar graph (D) show DAMGO-mediated potentiation of oEPSC amplitude in IPR neurons (n= 5 cells/ 5 mice); paired t-test, *$p < 0.05$.
(E) No significant effect of DAMGO on PPR was detected (n= 5 cells/ 5 mice); Wilcoxon test, $p > 0.05$.
Solid and open circles displaying individual neurons (D, E) represent data from male and female mice, respectively.

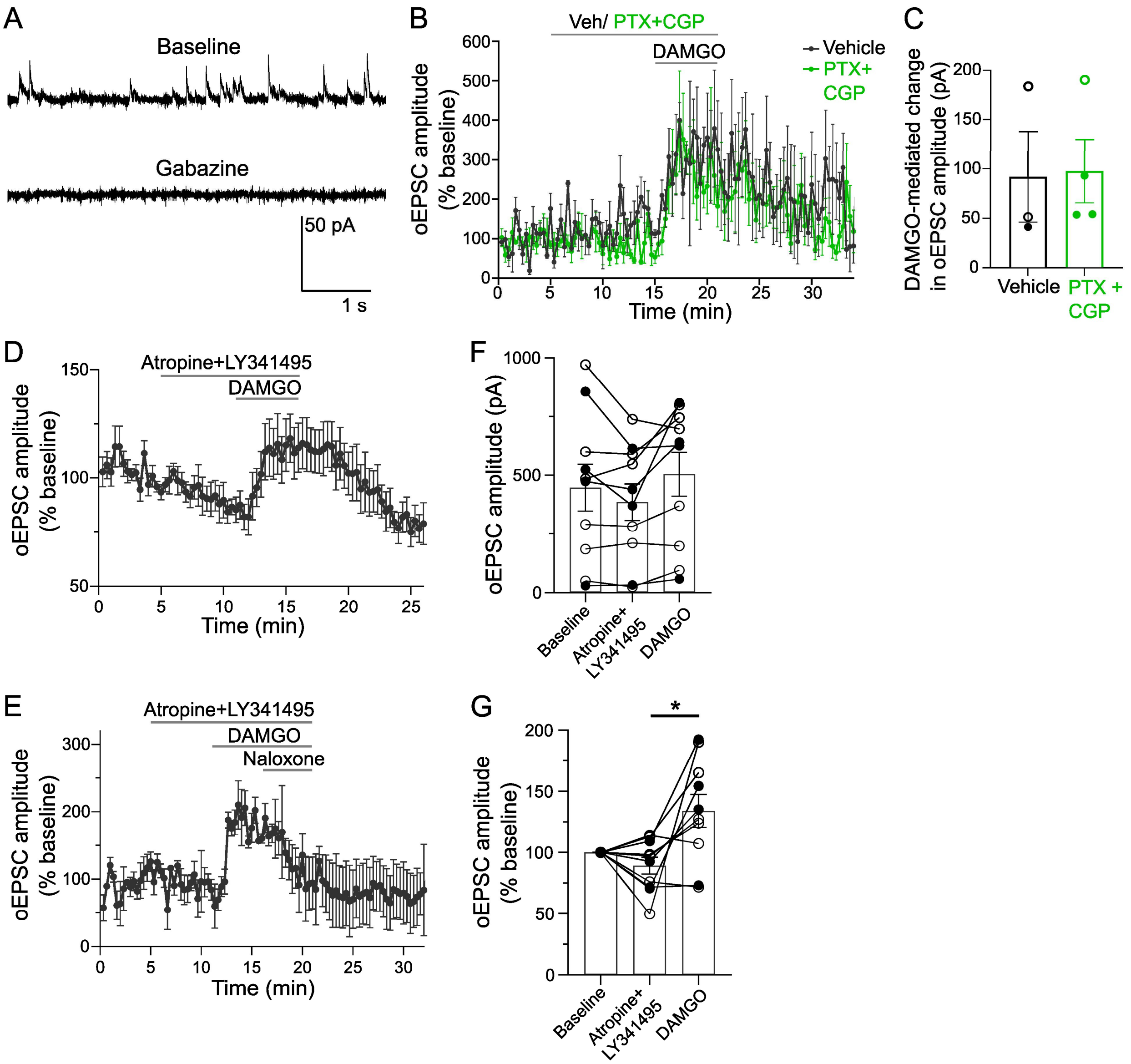

Figure S3 (related to Figure 3). MOR potentiation of excitatory transmission persists in the presence of GABA receptor blockers, as well as muscarinic receptor and mGluR blockers

(A) Example trace showing sIPSCs in an IPR neuron are blocked by gabazine (5 μM).

(B and C) Averaged time-trace (B) and bar graph (C) showing DAMGO (1 μM) potentiation of oEPSC amplitude (ChR2 expressed in MHb of MOR-Cre mice) persisted in the presence of PTX (50 μM) plus CGP (2 μM) (n= 4 cells/ 3 mice) or vehicle (n= 3 cells/ 3 mice); unpaired t-test, p> 0.05.

(D and E) DAMGO (1 μM) potentiation of oEPSC amplitude (ChR2 expressed in MHb of MOR-Cre mice) persisted in the presence of atropine (10 μM) plus LY-341495 (1 μM) (D, n= 7 cells/ 5 mice), and reversed upon application of naloxone (5 μM) (E, n= 3 cells/ 3 mice).

(F) Effects of atropine + LY-341495 and DAMGO on oEPSC amplitude (n= 10 cells/ 5 mice); repeated-measures one-way ANOVA, $F_{1.43,12.9}$= 3.0, p= 0.09. DAMGO's facilitatory effects on oEPSC amplitude were observed in 7/10 cells.

(G) Normalized bar graph showing DAMGO mediated potentiation of oEPSC amplitude in the presence of atropine + LY-341495 (n= 10 cells/ 5 mice); t_9= 3.0, p= 0.01; *p< 0.05.

Solid and open circles displaying individual neurons (C, F, G) represent data from male and female mice, respectively.

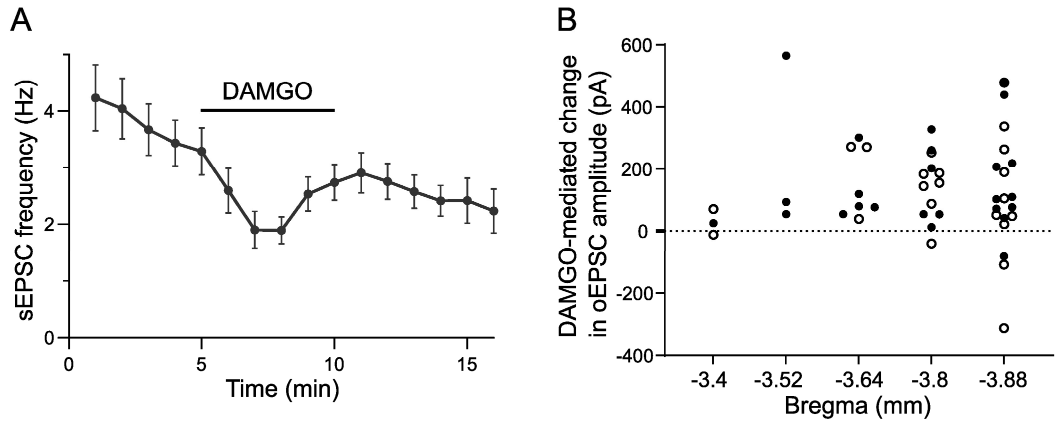

Figure S4. MOR activation canonically reduces spontaneous neurotransmission at HP synapses and inhibits evoked excitatory transmission in some caudal IPR cells

(A) Averaged time-trace of whole-cell recordings showing DAMGO (1 μM)-mediated transient inhibition of sEPSC frequency in IPR neurons (n= 58 cells/ 41 mice). This data is a more extensive analysis of MOR's effects on spontaneous excitatory transmission at HP synapses and represents all cells reported in this paper treated with DAMGO, except the cells already analyzed for sEPSC frequency in Figures 1J-1L and the cells pre-treated with TTX+4AP described in Figures 3C-3E. Note that there is variability in the genotypes and the pharmacological treatment that precede DAMGO application because this secondary analysis of sEPSC frequency averaged across a mixture of datasets.

(B) MOR potentiation of evoked excitatory transmission was observed across the rostral-caudal extent of IPR. However, a subset of IPR neurons in caudal IPR appeared to show a canonical inhibitory effect of MOR activation. These data are a representation of DAMGO responses from across the experiments shown in Figures 2, 3 and S3. Solid and open circles showing individual neurons represent data from male and female mice, respectively.

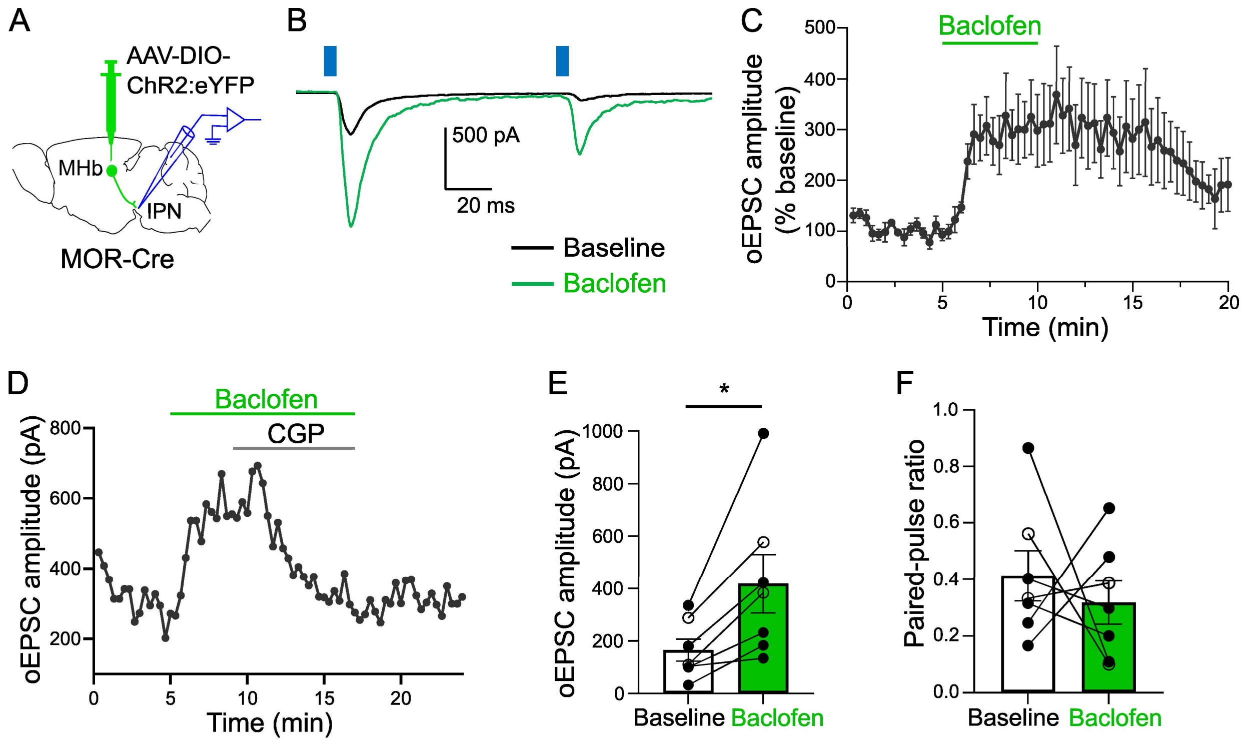

Figure S5. $GABA_B$ receptor activation can potentiate excitatory transmission at the MOR^+ HP synapse

(A) Experiment design showing patch-clamp electrophysiology in IPN following AAV injection into MHb of MOR-Cre mice.

(B) Example trace showing baclofen (5 µM) potentiation of oEPSC amplitude in an IPR neuron.

(C and D) Averaged time trace show baclofen-mediated potentiation in oEPSC amplitude (C, n= 6 cells/ 4 mice), which was reversed by the $GABA_B$ receptor antagonist CGP-55845 (2 µM) (D, n= 1 cell/ 1 mouse).

(E) Bar graph show baclofen-mediated change in oEPSC amplitude (n= 7 cells/ 4 mice); paired t-test, *p< 0.05.

(F) Baclofen mediated changes in PPR were variable (n= 7 cells/ 4 mice); paired t-test, p> 0.05.

Solid and open circles displaying individual neurons (E, F) represent data from male and female mice, respectively.
